# Multiplexed detection of various breast cancer cells by perfluorocarbon/quantum dot nanoemulsions conjugated with antibodies

**DOI:** 10.1186/s40580-014-0023-5

**Published:** 2014-07-08

**Authors:** Pan Kee Bae, Bong Hyun Chung

**Affiliations:** Bionanotechnology Research Center, Korea Research Institute of Bioscience and Biotechnology, Daejeon, 305-806 Korea

**Keywords:** Perfluorocarbon, Quantum dot, Breast cancer, Bimodal imaging

## Abstract

The effective targeting of cancer cell surface antigens is an attractive approach in cancer diagnosis and therapy. Multifunctional nanoprobes with cell-targeting specificity are likely to find important applications in bioanalysis, biomedicine, and clinical diagnosis. In this study, we have fabricated biocompatible perfluorocan/quantum dot nanoemulsions as bimodal imaging nanoprobes for the targeting of breast cancer cells. Perfluorocarbon/quantum dot nanoemulsions conjugated with monoclonal antibodies, as a type of bimodal imaging nanoprobe based on ^19^ F-MR and optical imaging, have been synthesized and applied for targeted imaging of three different breast cancer cells (SKBR3, MCF-7, MDA-MB 468), respectively. We have shown that the cancer-detection capabilities of antibody-conjugated PFC/QDs nanoemulsions could be successfully applied to target of various breast cancer cells. These modified PFC/QDs nanoemulsions were shown to target the cancer cell surface receptors specially. Conjugation of ligands to nanoemulsions targeting over-expressed cell surface receptors is a promising approach for targeted imaging to tumor cells. We further propose that the PFC/QDs nanoemulsions could be used in targeted imaging of breast cancer cells.

**Electronic supplementary material:**

The online version of this article (doi:10.1186/s40580-014-0023-5) contains supplementary material, which is available to authorized users.

## 1 Background

Medical imaging technologies have undergone explosive growth over the past few decades and now play a central role in clinical oncology. Targeted magnetic resonance imaging (MRI) has emerged as a promising diagnostic approach offering high resolution depictions of pathological anatomy and the detection of associated disease biomarkers [1-3]. Semiconductor quantum dots (QD) or organic fluorophores as luminescence probes for many biological and biomedical applications have long been used to visualize cell biology at many levels, from molecules to complete organisms [4-8]. Biologically bifunctional nanoparticles with unique MR and optical imaging capabilities are emerging as useful probes for biolabeling, tumour-targeting, disease diagnosis, cell-based therapy, targeted molecular imaging, optical sensing and biosensors, drug delivery, and the monitoring of therapeutic effects [9-13]. The multimodal imaging will allow clinicians to not only see where a tumour is located in the body, but also to visualize the expression and activity of specific molecules and biological processes that influence tumour behaviour and/or response to therapy.

Recently, many research groups in the world have made every effort to synthesize the nanoparticles that possess both fluorescent and magnetic properties. The combination of fluorescent and magnetic properties is a powerful tool allowing manipulation by magnetic fields and visualization/detection by fluorescence. The perfluorocarbon (PFC) has been explored for various medical applications, including liquid lung ventilation [14], gastrointestinal contrast agent [15], and blood substitutes [16,17]. PFCs are a class of molecules that are highly useful for intracellular MRI tracer applications. While the ^19^ F isotope of fluorine has a natural abundance of near 100%, the biological presence is virtually zero. Therefore, the ^19^ F molecule in PFC nanoemulsions has excellent properties for MR spectroscopy and imaging without a surrounding signal from endogenous fluorine [18-20]. Because of their size-tunable emission spectra, broad absorption spectra, high quantum yields, and exceptional resistance to photo and chemical degradation, QDs are currently being investigated for many biological and biomedical applications as luminescence probes [21-25].

In this context, we developed the fabrication of PFC/QD nanoemulsions as bimodal imaging nanoprobes for the targeting of breast cancer cells. Also, we have shown that the cancer-detection capabilities of antibody-conjugated PFC/QD nanoemulsions could be successfully applied to target of various breast cancer cells. We further propose that the PFC/QD nanoemulsions could be used in targeted imaging of breast cancer cells.

## 2 Methods

### 2.1. Materials

Perfluorooctylbromide (PFOB), 1H,1H,2H,2H-perfluorode-canethiol (PFDT), 3-(4,5-dimethylthiazol-2-yl)-2,5-diphenyltetrazolium bromide (MTT), and cholesterol were supplied by Sigma-Aldrich (St. Louis, MO). Perfluoro-15-crown ether (PFCE) was purchased from SynQuest Laboratories (Alachua, FL). 3-(N-succinimidyloxyglutaryl) aminopropyl polyethyleneglycol-carbamyldistearoyl phosphatidyl-ethanolamine (DSPE-PEG_3400_-NHS) was supplied by NOF Co. (Tokyo, Japan) and CdSe/ZnS QDs was obtained from Evident Technologies (Troy, NY). L-α-phosphatidylcholine (lecithin, 95%, chicken egg) was purchased from Avanti Polar Lipids (Alabaster, AL). Antibodies were from following sources: anti-human EGF1R (AbD serotec, Oxford, UK), anti-human ErbB2 and anti-human IGF1R (R&D Systems, Inc., Minneapolis, MN). All compounds for the cell culture were supplied by Invitrogen (Carlsbad, CA).

### 2.2. Preparation of PFC/QD nanoemulsions

To fabricate PFC/QD nanoemulsions, two PFC materials [PFCE and PFOB] and two different colored QDs (emitting at 525, 606 nm) were used. For conventional hydrophobic ligand-capped CdSe/ZnS QDs to be compatible with PFC liquids, the hydrophobic ligands of QDs should be exchanged with PFDT. A 7 mg CdSe/ZnS QDs dispersed in toluene was mixed with 34 g PFC liquids containing 1 ml PFOT and methanol. After vigorous mixing of the two-phase solutions for 24 h, the CdSe/ZnS QDs were partitioned into the PFC phase. The QDs in PFC were then washed three times with methanol to remove the excess ligands from the solution. The PFC liquids containing CdSe/ZnS QDs were emulsified in an aqueous solution using surfactant mixtures. A surfactant mixture composed of 78.5 mol% lecithin, 20 mol% cholesterol, and 1.5 mol% DSPE-PEG_3400_-NHS were dissolved in chloroform, and the organic solvent was evaporated using a rotary evaporator and a freezing dryer for 24 h. After dispersing the surfactant mixture into sterilized distilled water, the solution was sonicated. PFC/QD solution (40% v/v), the surfactant mixture (2% w/v) and phosphate buffered saline were mixed for 4 min using a homogenizer. The mixture was followed by microfluidization. A Microfluidizer M-110S (Microfluidics, Inc., Newton, MA) operating at a liquid pressure of approximately 20000 psi was used for all reported nanoemulsion preparations. The fabricated PFC/QDs nanoemulsions were separated using the size-exclusion column and were stored at 4°C.

### 2.3. Preparation of antibody conjugated PFC/QD nanoemulsions

For targeting the breast cancer cells, three different antibodies [anti-human EGF1R, anti-human ErbB2 and anti-human IGF1R] were conjugated to the PFC/QD nanoemulsions. Antibody-conjugated PFC/QD nanoemulsions were developed by linking the carboxyl groups on the surface of PFC/QD nanoemulsions with the amine groups in antibodies. To the solution containing PFC/QD nanoemulsions and antibodies, 1-ethyl-3-(3-dimethylamino propyl)carbodiimide (EDC) was added and permitted to react with the NHS ligand of PFC/QD nanoemulsions for 2 h at room temperature. To quench the reaction, add 2-mercaptoethanol to a final concentration of 10 mM. The antibody-coated PFC/QDs nanoemulsions were purified by size-exclusion with sepharose 4B column and concentrated using ultrafiltration for 30 min.

### 2.4. Characterization of PFC/QD nanoemulsions

The emission spectra of PFC/QD nanoemulsions were measured using a fluorescence spectrometer (LS 55, PerkinElmer Instruments, Wellesley, MA). The mean particle diameters and zeta (ζ)-potentials of the PFC/QD nanoemulsions were determined using a particle size analyzer (ELS-Z, Otsuka Electronics, Japan). Measurements were taken after diluting the nanoemulsion in water and equilibrating at room temperature for at least 30 minutes prior to each measurement. All measurements were taken at room temperature.

### 2.5. Cell viability assay

The cell viability was assessed by a modified MTT assay. Cell viability was measured for the following three cell lines: SKBR3, MCF-7, and MDA-MB 468 cells. These cell lines were obtained from the American Type Culture Collection (Rockville, MD). Human breast cancer cell lines SKBR3, MCF-7, and MDA-MB 468 were grown and maintained in each medium McCoy’s, MEM-α, RPMI1640 supplemented with 10% heat inactivated FBS, 50 IU ml^−1^ penicillin, and 50 μg ml^−1^ streptomycin, respectively. Each cell was plated into a 96-well plate (Corning Costar, Cambridge, MA) at 1 × 10^4^ cells/well. After incubation for 24 h, the medium was removed and the prepared PFC/QDs nanoemulsions diluted to several different concentrations (0, 2.5, 7.4, 22.2, 66.7, 200 μl ml^−1^) were poured into the wells. After another 24 h incubation period, the residual nanoemulsions were removed and an MTT solution at a concentration of 2.5 mg ml^−1^ in magnesium- and calcium-free phosphate-buffered saline was added to each well. The wells were then incubated in a humidified CO_2_ incubator at 37°C for 1.5 h. 100 μl of acidified isopropanol/10% Triton X-100 solution was then added and the plates were shaken to dissolve the formazan products. The absorbance at 570 nm was measured with a microplate reader (Bio-rad, Hercules, CA) The cell survival rate in the control wells without the PFC/QD solutions was considered as 100% cell survival.

### 2.6. In vitro fluorescence and ^19^ F-MR imaging of breast cancer cells

For fluorescence imaging, SKBR3, MCF-7, or MDA-MB 468 cells were incubated with 50 μl ml^−1^ antibody-conjugated PFC/QD nanoemulsions (ErbB2-PFCE/QD606, EGF1R-PFOB/QD525, IFG1R-PFOB/QD606) for 24 h at 37°C. After being washed with PBS, the labeled cells were fixed with Cytofix/Cytoperm solution and stained with DAPI in PBS. Fluorescence images were obtained on a Deltavision RT deconvolution microscope (Applied Precision Technologies, Issaquah, WA) using emission filters (525WB20, 600WB20, Omega Optical, Brattleboro, VT). All ^19^ F-MR imaging experiments of PFC/QD nanoemulsions were performed with a 4.7 T Bruker scanner (Biospec, Rheinstetten, Germany) using a double-tuned ^1^H/^19^F quadrature birdcage RF resonator. A ^19^ F-MR image was captured with a FLASH sequence (128 × 128 matrix; 30 × 30 mm^2^ FOV; 50 ms TR; 2.6 ms TE; 12 mm slice thickness).

### 2.7. Statistical analysis

The statistical evaluations of the experiments were performed by ANOVA analysis followed by a Newman-Keuls multiple comparison test.

## 3 Results and discussion

**Figure 1:**
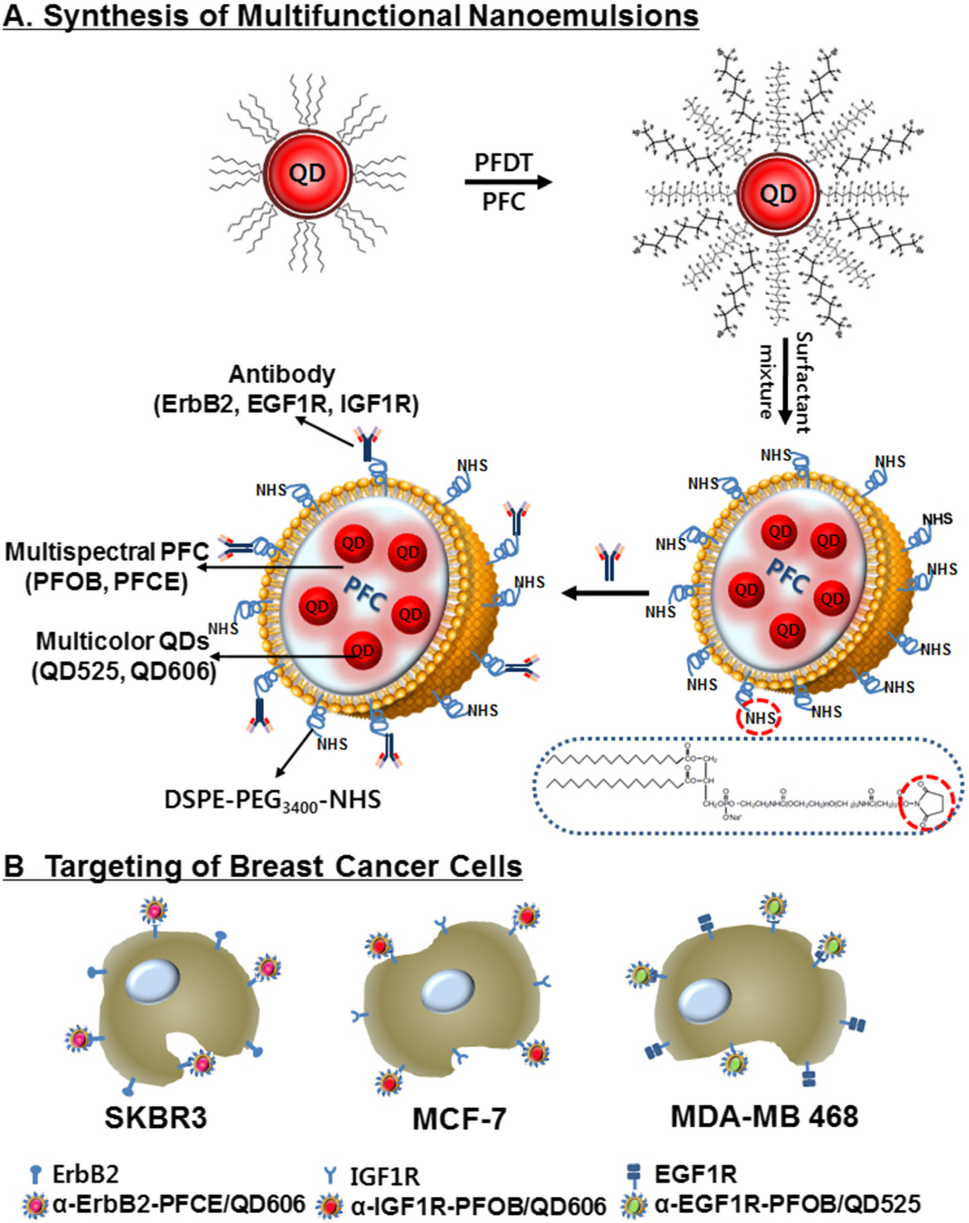
**A schematic representation of three different liquid PFC/QDs nanoemulsions (A) and their recognition of growth factor receptor in breast cancer cells (B).** The PFC core is surrounded by a lipid monolayer that is functionalized with DSPE-PEG_3400_-NHS to conjugate with three different antibodies.

**Figure 2:**
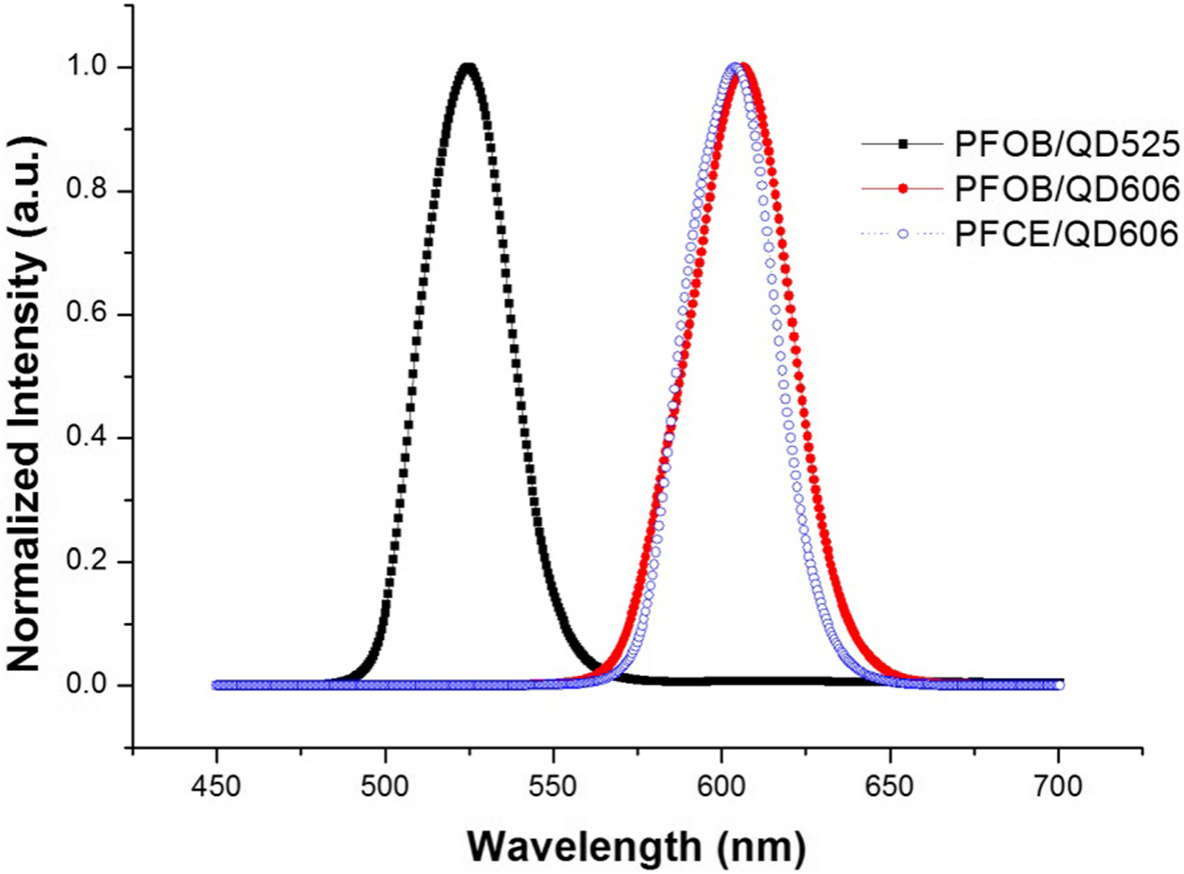
**Fluorescence spectra of the different three PFC/QDs nanoemulsions, excited at 390 nm.** Narrow emission bands (31–35 nm FWHM or full-width at half-maximum) indicate narrow particle size distributions.

**Figure 3:**
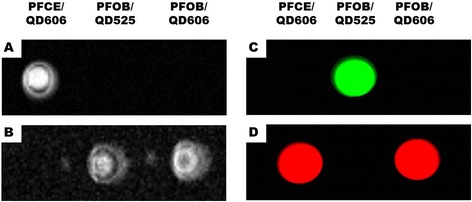
^**19**^ **F MR and fluorescence imaging of PFC/QDs nanoemulsions: A) PFCE-resonance selective MR image, B) PFOB-resonance selective MR image, C) image obtained by using a green filter, and D) image obtained by using a red filter.**

We have fabricated multifunctional PFC/QD nanoemulsion particles for molecular imaging and targeted breast cancer cells (Figure 1). The engineered PFC/QD nanoemulsions are composed of two PFC materials (PFCE and PFOB) and two different colored QDs (CdSe/ZnS 525, 606 nm). These nanoemulsions provide both ^19^ F-based MR and optical imaging capabilities. To increase the compatibility of hydrophobic QDs with PFC, tri-*n*-octylphosphineoxide (TOPO) coated QDs were exchanged with PFDT [26]. After vigorous stirring of the toluene-PFC mixture, the CdSe/ZnS QDs migrated from the toluene phase to the PFC phase. For PFC liquids containing CdSe/ZnS QDs to be useful as probes, for the examinations of biological specimens, three different PFC/QD (PFCE/QD606, PFOB/QD525, PFOB/QD606) nanoemulsions were emulsified in an aqueous solutions using surfactant mixtures. The fluorescence intensity and peak wavelength of these nanoemulsions was measured using a fluorescence spectrometer (Figure 2). The full-width-at-half-maximum (FWHM) values of these nanoemulsions are 31 – 35 nm, respectively. Because of their narrow and symmetric emission spectra, high-quality PFC/QD nanoemulsions are well suited to optical multiplexing. The zeta potential and average size of different PFC/QD nanoemulsions were measured by dynamic light scattering analysis (Table 1). To investigate the dual-mode imaging capabilities of PFC/QD nanoemulsions, we measured a ^19^ F-MR image of specific PFC using a 4.7 T Bruker scanner (Figure 3A,B) and an optical image of different color QDs using suitable wavelength optical filters (Figure 3C,D). The PFC/QD nanoemulsions containing two different PFC liquids (PFCE or PFOB) are discriminated by using the distinctly different spectrum of each PFC. These results suggest that the PFC/QD nanoemulsions could serve as more effective nanoprobes for the ^19^ F-MR imaging and fluorescence imaging of various breast cancer cells.

**Table 1:** **Summary of the optical and physicochemical properties of various PFC/QDs nanoemulsions**

**Sample**	**Emission peak (nm)**	**FWMH** ^**[a]**^	**Average size (nm)**	**Zeta potential (mV)**
PFOB/QD525	525	31	233	−21.27
PFOB/QD606	606	35	230	−20.54
PFCE/QD606	603	31	227	−18.03

^[a]^Full-width at half maximum.

**Figure 4:**
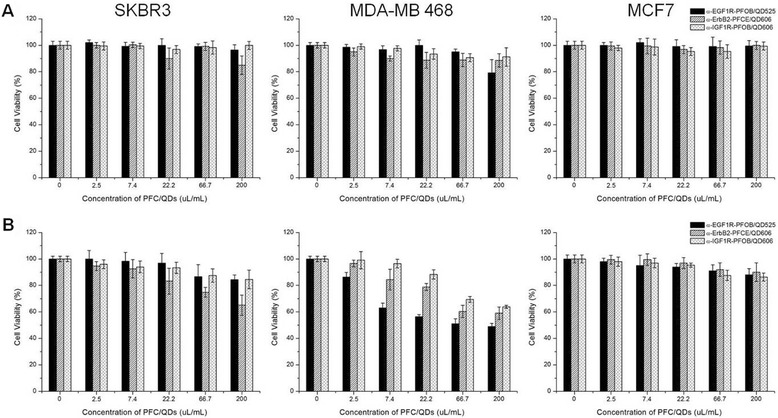
**Cell cytotoxicity for the different antibody-conjugated PFC/QDs nanoemulsions and different cell types, incubated at 37°C for 24 h (A) and 48 h (B).** Three different nanoemulsions are tested on the cell viability for each cell type.

We further investigated whether the PFC/QD nanoemulsions could be used in targeted imaging of breast cancer cells. The PFC/QD nanoemulsions were conjugated with monoclonal antibodies that can target epidermal growth factor receptors and other growth factor receptor: EGF1R (epidermal growth factor-1 receptor), ErbB2 (also called Her2 and Neu, epidermal growth factor-2 receptor), and IGF1R (insulin-like growth factor-1 receptor) (Figure 1B). These receptors are often overexpressed on the surface of human breast cancer cell lines [SKBR3 (ErbB2-positive), MDA-MB 468 (EGF1R-positive), MCF-7 (IGF1R-positive)] [27-29]. They serve as important targets for selective cancer therapy. The expression or activation of human growth factor receptors are altered in many epithelial tumours and clinical studies indicate that they have important roles in tumour aetiology and progression. For targeting the breast cancer cells, three different monoclonal antibodies (α-EGF1R, α-ErbB2, and α-IGF1R) were reacted with NHS on the PFC/QD nanoemulsions for 2 h at room temperature, respectively. DSPE-PEG_3400_-NHS lipid which has a distal NHS group at the end of PFC/QD nanoemulsions reacts with a homing molecule. The homing molecules were conjugated at the end of the lipid, and they specifically bind to their receptors on the breast cancer cell surface. The antibody-coated PFC/QD nanoemulsions purified by size-exclusion chromatography with a sepharose 4B column and the excess amount of antibodies were removed (Additional file 1: Figure S1). We evaluated the cell damage caused by the PFC/QDs nanoemulsions for biological applications. We performed cell viability assay to determine the cytotoxicity of various PFC/QD nanoemulsions based on the extracellular nanoemulsions exposure levels in cells (Figure 4). Different doses (0, 2.5, 7.4, 22.2, 66.7, 200 μl ml^−1^) of these nanoemulsions were used for *in vitro* cytotoxicity tests. We used three cell types: SKBR3 cell, MCF-7 cell, and MDA-MB 468 cell for three PFC/QD nanoemulsions. The result showed that no differences in three type cells were observed for PFC/QD nanoemulsions at 24 h and 48 h. The tendencies of the cell viability at 24 h with PFC/QD nanoemulsions were almost the same. Treatment of SKBR3 and MDA-MB 468 with 22.2 - 200 μl ml^−1^ of antibody-conjugated PFC/QD nanoemulsions significantly decreased the cell viability with respect to control at 48 h. Within 48 h the cell viability in SKBR3 cells decreased from 92 ± 6% to 65 ± 7% at the α-ErbB2-PFCE/QD606 concentration of 7.4 − 200 μl ml^−1^. Also, for the α-EGF1R-PFOB/QD525 concentration of 2.5 − 200 μl ml^−1^ the viability of MDA-MB 468 cells at 48 h decreased from 86 ± 3% to 49 ± 2%. There were no significant changes in cell viability for these nanoemulsions in MFC7 cells. Since QDs may slowly release the toxic Cd^2+^ or Se^2−^ ions into the solution, the particles must be as inert as possible for any in vitro application. The toxic of QDs not only depends on the concentration of free Cd^2+^ ions but also depends on whether the particles are ingested by a cell and where they are stored. The release of Cd^2+^ from the particles’ surface can be reduced by employing core/shell particles or the coating of the particles with silica, polymer, or liposome.

**Figure 5:**
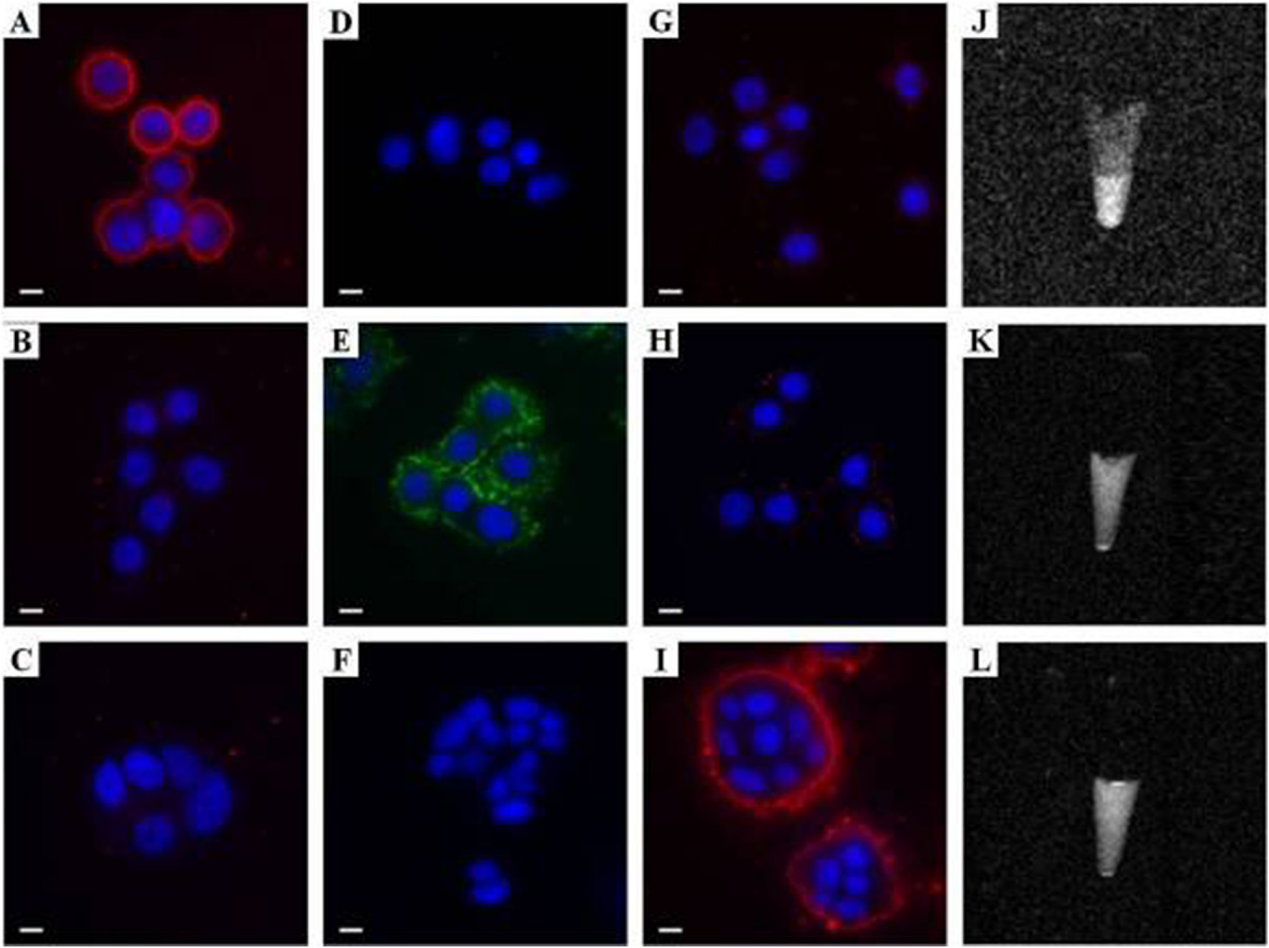
**Luminescence (A-I) and** ^**19**^ **F MR (J-L) images of cultured SKBR3 (A, D, G, J), MDA-MB 468 (B, E, H, K), and MCF-7 (C, F, I, L) cells as incubated with α-ErbB2-PFCE/QD606 (A-C, J), α-EGF1R-PFOB/QD525 (D-F, K) and α-IGF1R-PFOB/QD606 (G-I, L).** The QDs (green and red) and the DAPI-stained nuclei (blue) were recorded with Deltavision RT deconvolution microscope. The modified PFC/QDs nanoemulsions are shown in green and red, and the DAPI-stained nuclei are shown in blue. Scale Bars: 10 μm.

To investigate the targeting specificity, each breast cancer cell line was incubated with three different antibody-conjugated PFC/QD nanoemulsions (α-ErbB2-PFCE/QD606, α-EGF1R-PFOB/QD525, and α-IGF1R-PFOB/QD606). Fluorescence imagings were obtained on a Deltavision RT deconvolution microscope. As shown in Figure 4, the fluorescence of α-ErbB2-PFCE/QD606 nanoemulsions was only observed in the ErbB2-positive SKBR3 breast cancer cells (Figure 5A). MDA-MB 468 and MCF-7 cells showed only slight fluorescence signals with α-ErbB2-PFCE/QD606 nanoemulsions (Figures 5B,C). The attachment of α-ErbB2-PFCE/QD606 onto the SKBR3 cells suggests that there is a specific interaction between the α-ErbB2 that bound to PFC/QDs and ErbB2. Also, α-EGF1R-PFOB/QD525 and α-IGF1R-PFOB/QD606 nanoemulsions were targeted to the MDA-MB 468 and MCF-7 cells, respectively (Figure 5D-I). Also, the ^19^ F-based MR images for the specific targeting of each antibody-conjugated PFC/QD nanoemulsion in various breast cancer cells are shown (Figure 5J-L). These results indicate that antibody-PFC/QD nanoemulsions selectively bind to the target-protein. Therefore, the modified PFC/QD can act as a useful optical and ^19^ F-MR imaging agent for the diagnosis and targeting of breast cancer cells.

## 4 Conclusion

In conclusion, the present study describes a novel approach for detecting the various breast cancer cells with the antibody-conjugated PFC/QD nanoemulsions as a type of bimodal imaging nanoprobe with unique MR and optical imaging capabilities. It is believed that this approach will provide a very promising tool for the diagnosis of breast cancer. Different PFC/QD nanoemulsions can be conjugated to different antibodies, each targeted to specific proteins. The specific spectra of multiple PFC/QD targeted to different tissue proteins can then be simultaneously detected and quantified on one sample. They also have enhanced photostability, allowing the emission of fluorescent light over a length of time without a brisk decrease in emission, and the strength of their fluorescence means that low-level proteins can also be detected, thereby increasing diagnostic sensitivity [30-33]. PFC/QD nanoemulsions have great capacity as an efficient nanoprobe for targeting breast cancer cells. Furthermore, these nanoprobes have potential in a wider variety of novel applications that are related to anti-receptor therapy in cancer, molecular targeted approach, and modulated drug delivery.

## Additional file

Additional file 1: Figure S1. Gel permeation chromatogram of antibody-conjugated PFC/QDs nanoemulsions. The antibody-conjugated PFC/QDs nanoemulsions were separated by the sepharose 4B column. ▼, antibody-conjugated nanoemulsions; *, unreacted antibody.
