# Supplementary material for: Multiplexed detection of various breast cancer cells by perfluorocarbon/quantum dot nanoemulsions conjugated with antibodies
Source: Nano Converg. 2014 Jul 8;1(1):23. doi: 10.1186/s40580-014-0023-5 (PMC5270992; doi:10.1186/s40580-014-0023-5)
Supplement: Additional file 1: Figure S1. — Gel permeation chromatogram of antibody-conjugated PFC/QDs nanoemulsions. The antibody-conjugated PFC/QDs nanoemulsions were separated by the sepharose 4B column. ▼, antibody-conjugated nanoemulsions; *, unreacted antibody. [file 40580_2014_23_MOESM1_ESM.docx]

Supporting Information

Multiplexed detection of various breast cancer cells by perfluorocarbon/quantum dot nanoemulsions conjugated with antibodies

Pan Kee Bae, Bong Hyun Chung*

Bionanotechnology Research Center, Korea Research Institute of Bioscience and Biotechnology, Daejeon 305-806, Korea

* CORRESPONDING AUTHOR EMAIL ADDRESS: [chungbh@kribb.re.kr](mailto:chungbh@kribb.re.kr)

Tel) +82-42-860-4442

Fax) +82-42-879-8594

Pan Kee Bae : bpkee@kribb.re.kr


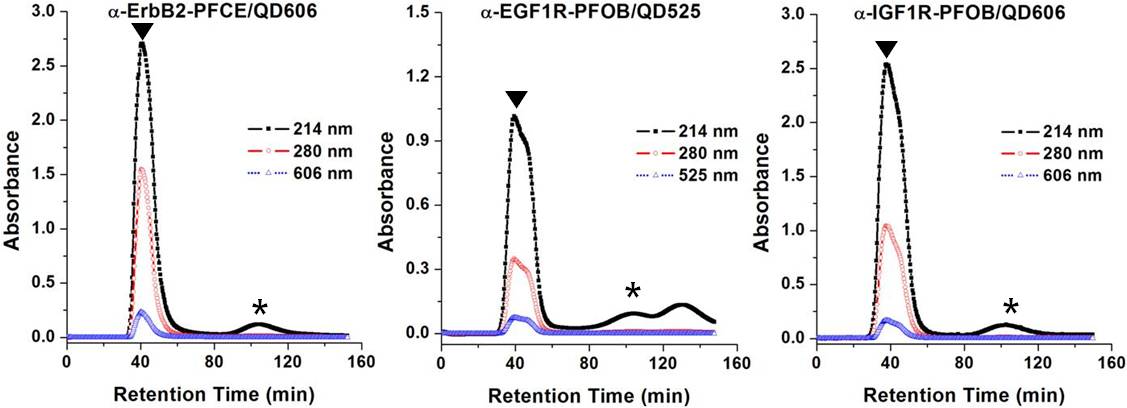


Figure S1. Gel permeation chromatogram of antibody-conjugated PFC/QDs nanoemulsions. The antibody-conjugated PFC/QDs nanoemulsions were separated by the sepharose 4B column. ▼, antibody-conjugated nanoemulsions; *, unreacted antibody.
